# Voluminous paraumbilical hernia containing the pancreas – An unusual cause of acute pancreatitis: A case report

**DOI:** 10.1016/j.amsu.2019.07.017

**Published:** 2019-07-10

**Authors:** Iman Komaei, Giuseppe La Malfa, Cristina Damiano, Federica Sarra, Gabriele Cassaro, Adalberto Barbera, Marcello Bartolotta, Giuseppe Currò, Giuseppe Navarra

**Affiliations:** Department of Human Pathology of Adult and Evolutive Age, General Surgery Unit, Surgical Oncology Division, University Hospital of Messina, Via Consolare Valeria 1, 98125, Messina, Italy

**Keywords:** Paraumbilical hernia, Intestinal obstruction, Emergency laparotomy, Acute pancreatitis, Case report

## Abstract

**Introduction:**

The paraumbilical hernia sac often contains the omentum, the small bowel, and less commonly the colon. The herniation of the pancreas through a paraumbilical hernia is extremely rare and has been reported only by two cases in the literature; moreover, acute pancreatitis secondary to this condition is a particularly unusual event.

**Case report:**

We present a very unusual case of a 67-year-old female patient with a voluminous paraumbilical hernia containing the pancreas, complicated by acute pancreatitis. Laboratory data revealed an elevation of the pancreatic enzymes. An intravenous contrast-enhanced computed tomography (CT) scan of the abdomen demonstrated a large hernia sac containing multiple viscera, including the pancreas. The patient underwent emergency laparotomy with a diagnosis of intestinal obstruction.

**Conclusion:**

The clinicians should consider this rare condition in the differential diagnosis of patients presenting with large paraumbilical hernias associated with classical symptoms of acute pancreatitis, particularly in the absence of typical risk factors for pancreatitis. An intravenous contrast-enhanced abdominal CT scan should be performed immediately in these patients. We recommend the patients and the surgeons to consider prompt surgical repair for paraumbilical hernias to avoid further complications and the higher incidence of morbidity and mortality associated with emergency surgeries.

## Highlights

• Paraumbilical hernia sac often contains omentum, small bowel, and seldom colon.
• Herniation of pancreas through a paraumbilical hernia is an extremely rare event.
• Pancreatitis linked to paraumbilical hernias is associated with high mortality.
• Prompt surgical repair of umbilical hernias in high risk patients is recommended.

## 1 Introduction

Umbilical hernias constitute around 10% of abdominal wall hernias [1]. Indirect umbilical hernias, also known as paraumbilical hernias, protrude above or below the umbilicus and are the most common type of umbilical hernias in adults, more frequently occurring in women. Paraumbilical hernias usually occur as result of an acquired abdominal wall defect associated with weakened peri-umbilical fascia and conditions that lead to chronic elevation of intra-abdominal pressure (e.g., obesity, multiple pregnancies, ascites, and large abdominal tumours) [2,3]. The content of the hernia sac is usually pre-peritoneal fat tissue, omentum, small bowel and seldom colon.

Herniation of the pancreas through a paraumbilical hernia is extremely rare, with only two cases previously reported in the literature [4,5]. Moreover, acute pancreatitis due to this condition is an exclusively rare event, as only a single case has ever been reported [4]. We report an extremely rare case of a 67-year-old female patient presenting with a voluminous paraumbilical hernia containing multiple viscera including the pancreas, complicated by acute pancreatitis. The current case report has been reported according to SCARE criteria [6].

## 2 Presentation of case

A 67-year-old, Caucasian, multiparous, pensioner woman walked into the emergency room (ER) with a 3-day history of severe epigastric pain radiating to the back, nausea, vomiting and a 5-year history of a large non-reducible paraumbilical hernia superior to the umbilicus. The hernia had gradually increased in size without causing obstructive symptoms; the reason why the patient had not sought prior medical assistance. She had no significant past medical and surgical history and was not on any medication. She denied drug allergies, tobacco, alcohol, and substance use. Her body mass index (BMI) was 24 kg/m^2^. The patient was eventually transferred from ER to our surgical unit for further investigations.

**Fig. 1:**
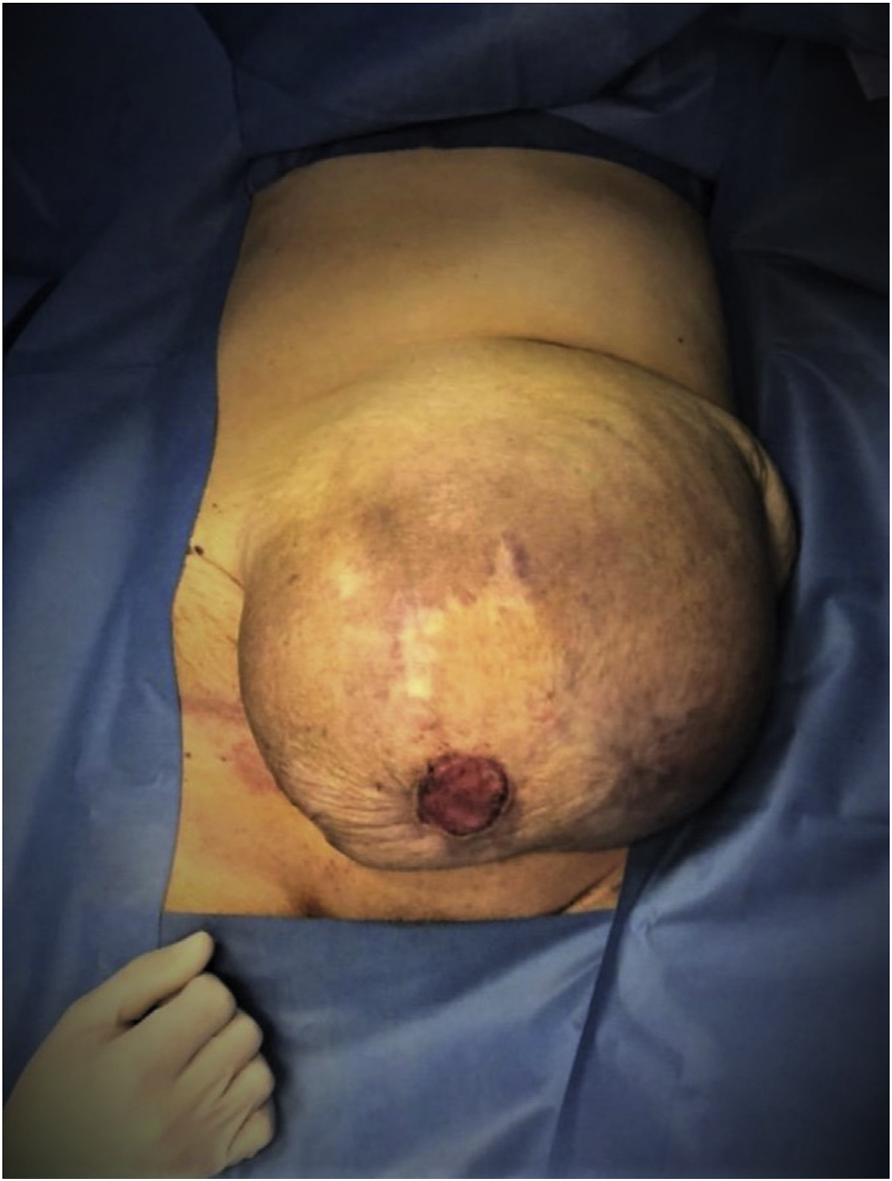
Massive, circumferential, tender, irreducible paraumbilical hernia with ulcerated areas of overlying skin.

On initial assessment, she appeared hypotensive, tachycardic, and tachypnoeic. On physical examination, a massive circumferential, tender, irreducible supraumbilical mass (transverse diameter of about 30 cm) with ulcerated areas of overlying skin was noted (Fig. 1).

**Fig. 2:**
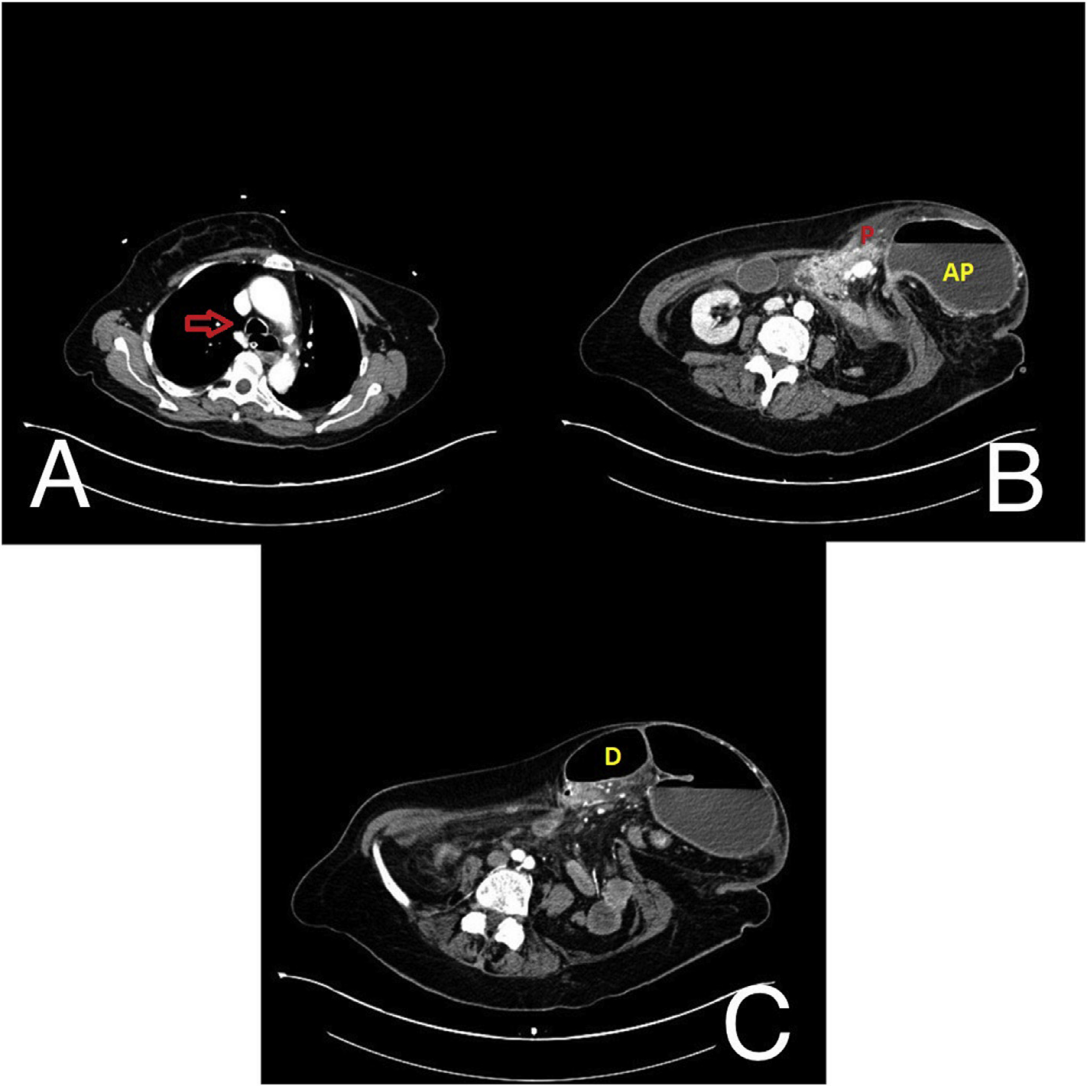
Pre-operative IV contrast-enhanced CT scan of the thorax and abdomen. Image A demonstrates the marked distension of the oesophageal lumen with air fluid levels (arrow). Image B demonstrates the voluminous paraumbilical hernia sac containing antro-pyloric portion of the stomach with air-fluid level (AP) and pancreas (P) protruding through an abdominal wall defect of about 6.5 cm. Image C demonstrates the markedly distended first portion of the duodenum (D) protruding into the hernia sac, pulling with it a portion of the pancreas.

Laboratory data revealed white blood cells 24 × 10^3^/mL, C-reactive protein 1809 nmol/L, pancreatic amylase 24150 nmol/s•L, serum lipase 47900 nmol/s•L, blood urea nitrogen 34 mmol/L, and creatinine 0.16 mmol/L. Other labs included normal liver function tests and normal bilirubin levels. The urinary catheter and nasogastric tube were placed. Urine output was poor and the nasogastric tube drained around 1500 ml of bilio-enteric material. An urgent intravenous (IV) contrast-enhanced computed tomography (CT) scan of the thorax and abdomen was performed. CT scan of the thorax demonstrated mild bilateral pleural effusion and marked distension of the oesophageal lumen with air fluid levels. Abdominal CT revealed a voluminous left paramedian supraumbilical hernia protruding through an abdominal wall defect of about 6,5 cm. Hernia sac contained loops of the small bowel and their mesentery, as well as the segments of ascending, transverse and descending colon. Additionally, the antro-pyloric portion of the stomach and the first portion of the duodenum with air-fluid levels were herniated through the sac, pulling with them a portion of the pancreas. The body and tail of the pancreas appeared oedematous with peri-pancreatic fluid collection and mesenteric stranding as a sign of glandular sufferance (Fig. 2). Neither gallbladder stones nor dilation of intra- and extra-hepatic bile ducts were reported.

**Fig. 3:**
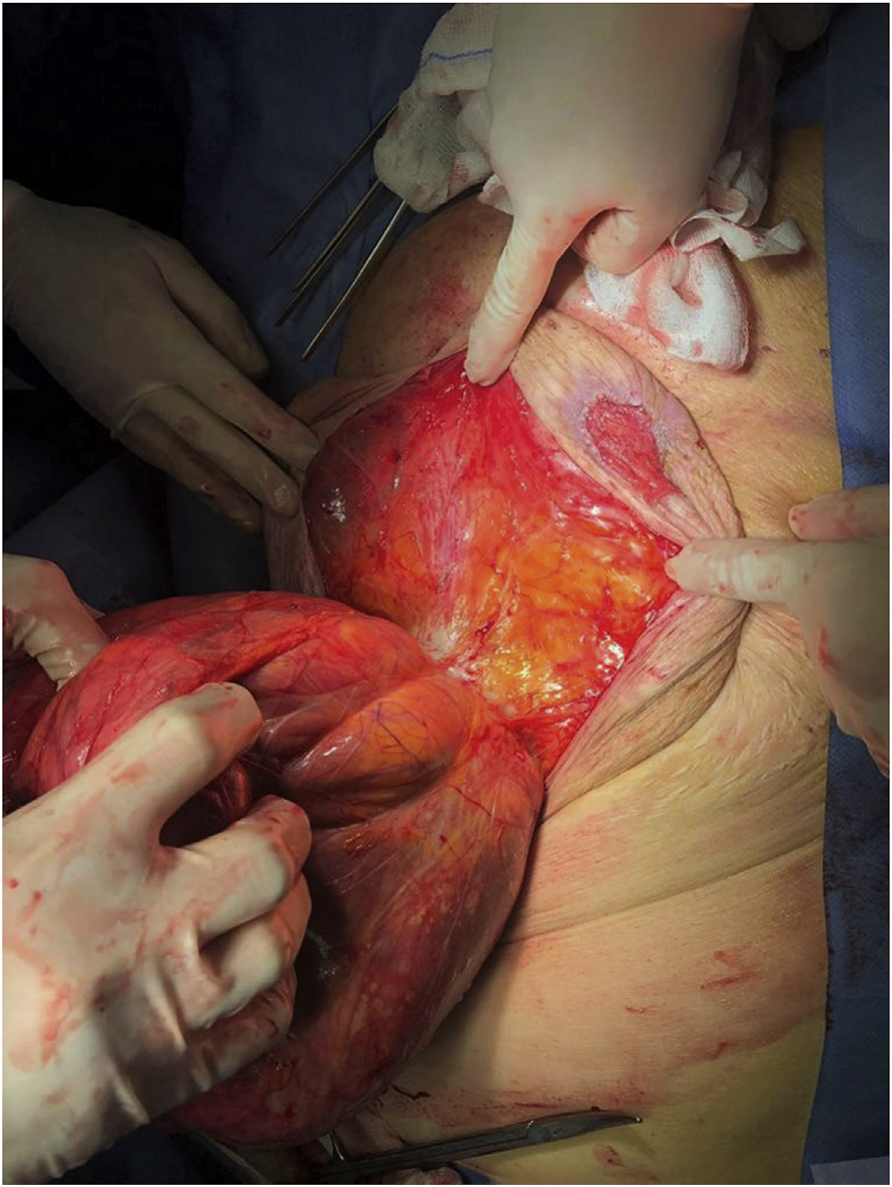
Isolated paraumbilical hernia sac protruding through the abdominal wall defect.

**Fig. 4:**
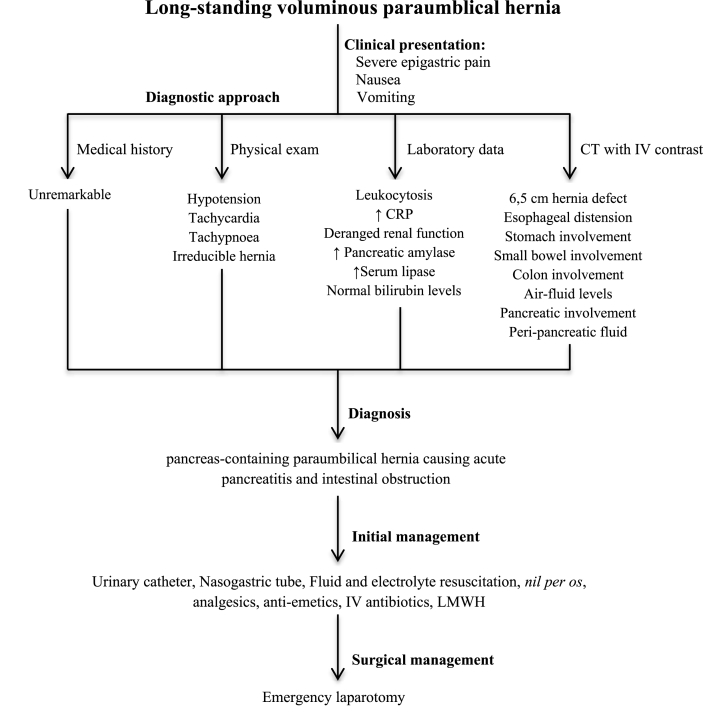
Schematic representation of the diagnostic and therapeutic approach to the presented patient with intestinal obstruction and acute pancreatitis due to the voluminous pancreas-containing paraumbilical hernia.

Following primary management with fluid and electrolyte resuscitation, *nil per os*, analgesics, anti-emetics, IV antibiotics, and venous thromboembolism prophylaxis with low molecular weight heparin (LMWH), the patient was transferred to the operating theatre on the same day with a diagnosis of intestinal obstruction in order to undergo an emergency laparotomy. The patient was placed in a supine position and general anaesthesia was induced. The surgery was performed by senior general surgeon G.L., experienced in emergency surgery and abdominal wall hernia repair. Following a midline incision, the voluminous hernia sac was identified, isolated, and opened (Fig. 3). Contents included the omentum, small bowel, large bowel, stomach, duodenum and a portion of the pancreas. After a thorough adhesiolysis, omentectomy was performed, the hernia sac was resected and the hernia contents were placed back into the abdominal cavity and the peritoneum was closed. A direct abdominal fascial closure was performed; however, no surgical mesh was implanted due to the high risk of post-operative mesh infection and the critical state of the patient. The ulcerated and excessive portions of the skin were resected and the fat and skin were directly sutured. Post-operatively, the patient was transferred to the intensive care unit. She was very unstable requiring vasopressors to maintain her blood pressure and her renal and respiratory functions gradually deteriorated. She developed multi-organ failure (MOF) and unfortunately passed away after 20 days. Fig. 4 demonstrates the schematic representation of the diagnostic and therapeutic approach to the presented patient.

## 3 Discussion

The possibility of migration of the pancreas through paraumbilical hernias is extremely rare. To our knowledge, there have only been two previous cases in the literature reporting a pancreas-containing paraumbilical hernia [4,5]. In general, trans-hernial migration of the pancreas is an unlikely event owing to the retroperitoneal location of the organ and its fixation by the ligament of Treitz. The involvement of the pancreas in the hernia sac is possible only if the transverse mesocolon is loose and similarly herniated through the hernia sac, as in our patient [7]. In such cases, the stretching of the transverse mesocolon may allow the mobilization of the pancreas after the lengthening of its posterior adhering fascia [4,8]. Acute pancreatitis as a complication of this phenomenon is even rarer and has been previously reported only in one patient [4].

Despite the challenges to definitively establish that pancreatic herniation was the sole aetiology of pancreatitis in our patient, other common causes such as gallstones, alcohol use, obesity, and hypercalcemia were excluded. Symptoms primarily consisted of pain localized to the epigastrium with radiation to the back, nausea, and vomiting. The diagnosis was confirmed by significant serum lipase and pancreatic amylase elevation and imaging evidence of pancreatic migration through hernia sac with inflammatory changes suggestive of pancreatitis (oedematous pancreatic tissue, peri-pancreatic fluid collection, mesenteric stranding).

Four possible mechanisms have been thought to be responsible for a hernia-associated pancreatitis: (1) the recurrent parenchymal trauma due to repetitive trans-hernial sliding of the organ; (2) ischemic insult as a result of intermittent stretching and traction of the vascular pedicle due to repetitive migration of the pancreas into the hernia sac; (3) volvulus or intermittent folding of the pancreatic duct leading to obstruction of pancreatic secretions, intraductal hypertension, and the resultant inflammation; and (4) Anoxic injury induced by Incarceration of the pancreas [4,9,10].

Due to the paucity of reported cases and experience, the optimal management of acute pancreatitis secondary to pancreatic herniation is not well established. Although due to the elevated risks, avoidance of an urgent surgical approach and a primary conservative management (e.g., IV fluid and electrolyte support, *nil per os*, IV antibiotic therapy, and analgesic control) is preferred, however, severe cases unresponsive to medical therapy, or cases involving perforation, or incarceration such as ours, require immediate surgical management [11]. Thus, the decision to perform a surgical repair should be considered on a case by case basis and following a thorough risk-benefit ratio assessment.

## 4 Conclusion

Although extremely rare, patients with large paraumbilical hernias may present with intermittent episodes of acute pancreatitis. The clinicians should consider this rare condition in differential diagnosis of patients presenting with recurrent episodes of epigastric pain, nausea, and vomiting associated with large paraumbilical hernias, particularly in the absence of typical risk factors for pancreatitis. An IV contrast-enhanced abdominal CT scan should be performed immediately in these patients. We recommend the patients and the surgeons to consider prompt surgical repair for paraumbilical hernias, particularly in high-risk patients, to avoid the risk of incarceration and strangulation and the higher incidence of morbidity and mortality associated with emergency surgeries.

## Patient consent

Written informed consent was obtained from the patient for publication of this case report and accompanying images. A copy of the written consent is available for review by the Editor-in-Chief of this journal on request.

## Provenance and peer review

Not commissioned, externally peer reviewed.

## Declarations of interest

None.

## Ethical approval

N/A

## Funding sources

None.

## Author contribution

-Iman Komaei: Performed the surgery, designed the study and wrote the first draft of the manuscript.

-Giuseppe La Malfa: Performed the surgery, revised the manuscript and made the final approval of the version to be submitted.

-Cristina Damiano: Performed the surgery, contributed to acquisition, validation, and visualization of the data.

-Federica Sarra: Contributed to acquisition, validation, and visualization of data.

-Gabriele Cassaro: Contributed to acquisition, validation, and visualization of data.

-Adalberto Barbera: Designed the study and helped to write the first draft of the manuscript.

-Marcello Bartolotta: Designed the study and helped to write the first draft of the manuscript.

-Giuseppe Currò: Revised the manuscript and made the final approval of the version to be submitted.

-Giuseppe Navarra: Revised the manuscript and made the final approval of the version to be submitted.

## Conflicts of interest

Iman Komaei, Giuseppe La Malfa, Cristina Damiano, Federica Sarra, Gabriele Cassaro, Adalberto Barbera, Marcello Bartolotta, Giuseppe Currò, and Giuseppe Navarra declare that they have no conflicts of interests or disclosures.

## Research registration unique identifying number (UIN)

Name of the registry: N/A.

Unique Identifying number or registration ID: N/A.

Hyperlink to the registration (must be publicly accessible): N/A.

## Guarantor

Iman Komaei
